# Prediction of body fat loss in relation to change in nutrient intake among housewives participating in the MyBFF@home study

**DOI:** 10.1186/s12905-018-0594-0

**Published:** 2018-07-19

**Authors:** Fatimah Othman, Noor Safiza Mohamad Nor, Geeta Appannah, Nor Azian Mohd Zaki, Rashidah Ambak, Azahadi Omar, Mansor Fazliana, Ruhaya Salleh, Barakatun Nisak Mohd Yusof, Norliza Muksan, Tahir Aris

**Affiliations:** 1 Centre for Nutrition Epidemiology Research, Institute for Public Health, National Institutes of Health, Ministry of Health Malaysia, Jalan Bangsar, 50590 Kuala Lumpur, Malaysia; 2 Department of Nutrition and Dietetics, Faculty of Medicine and Health Sciences, Universiti Putra Malaysia, Serdang, Malaysia; 3 Diabetes and Endocrine Unit, Cardiovascular, Diabetes and Nutrition Centre, Institute for Medical Research, Kuala Lumpur, Malaysia; 4 Klinik Kesihatan Rasa, Hulu Selangor, Malaysia

**Keywords:** Weight loss, Nutrient, Diet, body fat loss, individualised dietary intervention

## Abstract

**Background:**

Diet compositions are likely to be one of the influential factors for body fat deposition. The aim of this paper was to determine the nutrient changes and its association to body fat loss among the overweight and obese housewives in the MyBFF@home study.

**Methods:**

Data of participants in the MyBFF@home study (intervention and control groups) were analysed. Participants in the intervention group received personalised dietary counselling consisted of reduced calorie diet 1200–1500 kcal/day, while the control group was assigned to receive women’s health seminars. The dietary assessment was done during the intervention phase at baseline, 1 month (m), 2 m, 3 m and 6 m using a 3-day food diary. Body fat was measured using a bioelectrical impedance analyser (In-body 720) at baseline and at the end of the intervention phase. The mean differences of nutrient intake and body compositions during the intervention phase were measured with paired t-test. The changes in body fat and nutrients intake were calculated by subtracting baseline measurements from those taken at 6 months. Multiple linear regression analysis was conducted to determine the extent to which the changes in each gram of nutrients per 1000 kcal were predictive of changes in body fat mass.

**Results:**

There were significant reductions in energy, all macronutrients, dietary fibre, calcium and iron intake in both study groups after the intervention phase (*p* <  0.05). In the intervention group, body fat loss increased with the reduction of each gram of carbohydrate, protein and fat per 1000 kcal, (*p* <  0.05), and decreased with the reduction of each gram of calcium and fibre intake per 1000 kcal (*p* <  0.05). In the control group, body fat loss increased with the reduction of each gram fat per 1000 kcal (*p* <  0.05) and decreased with the reduction of each gram iron per 1000 kcal.

**Conclusion:**

Changes in the intake of various nutrients have different effects on body fat loss between the intervention and control group.

## Background

The obesity epidemic is one of the most significant health challenges in the world. In Malaysia, overweight and obesity have affected 30.0 and 36.2% of Malaysian adults respectively. A recent survey showed that women had a higher prevalence of obesity compared to men in Malaysia [1]. Obesity increases and aggravates the risk of cardiovascular disease, diabetes and other conditions. Furthermore, a higher number of women compared to men were reported to have known hypertension and diabetes as reported in the Malaysia National Health Morbidity Survey, 2015 [1]. Accordingly, women are more susceptible to develop weight-related non-communicable diseases such as diabetes and hypertension [2, 3].

It is clearly known that the positive balance between energy intake and energy expenditure is a cause for weight gain. Lifestyle modifications including weight loss diet and physical activity are among the therapeutic interventions that bring health benefits although such approaches bring down modest weight [4]. Weight can be stored as lean mass or fat mass and understanding causes that favour fat mass deposition could help in the prevention of obesity.

Diet compositions may be influential factors on body fat deposition. Previous studies assessing the correlation of obesity with particular nutrients such as carbohydrate and fat have shown various findings. Observational studies exploring diet composition and obesity were inconclusive [5]. While some studies presented an association between higher dietary fat and lower carbohydrate diet with body fat mass [6–8] others have not [9, 10]. It is also difficult to link whether the effect of dietary composition independent of total calorie intake is associated with adiposity [11], as the diet manipulations were regularly confounded by metabolic, physiological and environmental differences [12].

The effects of the macronutrients and other nutrients within the usual diet composition in free living-subjects remain unclear. Therefore, the present study aimed to predict the body fat loss in relation to change in nutrient intake among obese women both in the intervention and the control groups. To control the influence of energy intake as the nutrient intake increases when the food intake is increased, a standard measure of change in nutrients intake per 1000 kcal was used in the analysis. By identifying the relevant nutrient intake that influences changes in body fat, this study adds to the existing evidence and can contribute towards obesity management and prevention.

## Methods

The methodology of this quasi-experimental study was reported by Mohamad Nor et al. [13]. The participants of the MyBFF@home were obese housewives with lower socioeconomic status. Physical, anthropometric and clinical assessments were conducted per study protocol as described elsewhere [13, 14]. Data collection took place from 2014 to 2015. In the present study, 328 eligible participants were grouped into an intervention group (*n* = 169) and control group (*n* = 159). The intervention group received a lifestyle intervention consisting of a diet, physical activity and self-monitoring behaviour packages up to 6 months.

In the diet components, three-day food diaries (weekend and two weekdays) had to be filled out once a month throughout the intervention phase. The food diaries were returned to dietician/ nutritionist at each visit and it will be reviewed together with the subjects. The records were checked for discrepancies and omissions including cooking method, added ingredients in cooking, food brand, and portion size to ensure the validity of the records. Nutrient intakes were analysed using Nutritionist Pro TM version 2.4 (First Data Bank, The Hearst Corp, NY USA).

The 24-h diet recall was obtained at the baseline and during follow up for the basis of dietary counselling. The individualised dietary counselling consisted of portion control to achieve a low calorie-diet of 1200–1500 kcal/day with 50–55% carbohydrate, 25–30% fat, 15–20% protein, and 20 – 30 g fibre diet [15, 16]. This was prescribed based on the reported 24-h diet recall and energy needs that were calculated using quick method [16]. Other dietary behaviour skills including reading a food label, cues to eat management, eating outside were as explained in the study protocol. Apart from the individualised dietary approach, a group counselling involving sharing on weight loss experience, and positive actions were conducted to enhance diet adherence. The implementation of dietary counselling was based on the Trans theoretical Model (TTM) which has been described in the previous methodology [13].

Meanwhile, the control group was assigned to attend women’s health seminars organized as stipulated in the study protocol. For the dietary assessment, they were instructed to record a 3-day food diary monthly throughout the intervention period. However, no specific dietary interventions were given during the monthly follow up in the control group.

Participants were excluded from analysis if they did not have baseline data on nutrient intake, body fat, and body weight. The analyses were conducted using SPSS (IBM version 21). Variables were presented as mean and standard deviations as they were normally distributed. An independent t -test was used to compare the baseline nutrient intake and body fat measures. A paired t - test was used to determine the changes in body fat mass and dietary intake within study groups over the six-month intervention period. The changes in body fat and nutrient per 1000 kcal intake were calculated by subtracting baseline measurements from those taken at 6 months. Multiple Regression analysis using stepwise method was run to determine the extent to which the changes of nutrients per 1000 kcal were predictive of changes in body fat mass during the intervention period. The final model in the regression analysis included the baseline of body fat mass, age, household income, and baseline of nutrients intake per 1000 kcal to control the covariates.

## Results

Of the 328 housewives who initially participated in this study, 28 participants from the intervention group and 40 participants from the control group were excluded from the analysis due to incomplete data either from nutrient or body fat measures in the baseline. The final participants in the intervention group was (*n* = 137) and the control group (*n* = 118). Table 1 showed the participants nutrient intake and body fat measures at baseline. Dietary protein and calcium intake found to be significantly higher in the control compared to the intervention group (*p* < 0.05). There was no significant difference in other nutrient components between study groups. Body fat mass and body mass index of the intervention group was significantly higher than the control group (*p* <  0.05).

**Table 1:** Baseline nutrient intakes and body composition status

Variables	Intervention group (*n* = 137) Mean (sd)	Control group (*n* = 118) Mean (sd)	*p* value
Total energy intake (kcal / day)	1476.01 (508.83)	1569.23(517.87)	0.148
Carbohydrate (g/day) %	199.22 (68.63) (55%)	209.11 (78.29) (53%)	0.237
Protein (g /day) %	55.84 (20.89), (15%)	61.24 (20.83) (16%)	0.040^*^
Fat (g / day) %	49.35 (21.23) (30%)	53.31 (19.84) (31%)	0.127
Dietary fiber (g / day)	5.58 (3.83)	5.52 (3.17)	0.886
Calcium (mg / day)	415.39 (217.07)	478.03 (270.82)	0.042^*^
Folate (mg / day)	60.13 (56.37)	80.62 (42.25)	0.941
Iron (mg / day)	12.77 (6.63)	13.35 (6.24)	0.470
Vitamin C (mg / day)	62.53 (59.50)	71.13 (65.97)	0.275
Body fat (g)	34.87 (9.00)	32.79 (7.82)	0.037^*^
Body fat (%)	45.18 (4.86)	44.59 (4.79)	0.329
Body weight	75.99 (11.25)	72.61 (11.49)	0.018^*^

^*^Significant at *p* value < 0.05

Table 2 summarizes the body fat, BMI and nutrient changes over the six-month intervention period. At the end of the intervention program, both study groups experienced the body fat loss significantly (*p* <  0.05). However, greater reduction of body fat mass was observed in the intervention group (1.23 kg) compared to the control group (1.08 kg) (*p* = 0. 042).

**Table 2:** Body fat and nutrient changes during 6 months intervention phase

	Intervention group (*n* = 137)				Control group (*n* = 118)			
	Baseline	6 months	Mean Difference	*p* value	Baseline	6 months	Mean Difference	*p* value
Body fat (g)	34.87 (9.00)	33.64 (8.13)	1.23	< 0.001^**^	32.79 (7.82)	31.70 (8.12)	1.08	0.001^*^
Body fat (%)	45.18 (4.86)	44.39 (6.20)	0.80	0.070	44.59 (4.79)	44.04 (5.68)	0.55	0.130
BMI (kg /m^2^)	31.58 (4.11)	31.10 (4.20)	0.49	< 0.001^**^	30.92 (4.17)	30.49 (4.13)	0.43	< 0.001^**^
Total energy (kcal)	1476.00 (506.83)	1212.44 (415.93)	224.36	< 0.001^**^	1569.23 (517.87)	1344.87 (467.95)	224.36	< 0.001^**^
Carbohydrate (g/day)	198.21 (68.63)	169.72 (64.41)	28.50	< 0.001^**^	209.11 (78.29)	189.08 (73.50)	20.03	0.012^*^
Protein (g /day)	55.84 (20.89)	45.51 (14.11)	9.33	< 0.001^**^	61.24 (20.84)	53.26 (19.29)	7.98	< 0.001^**^
Total Fat (g / day)	49.35 (21.23)	38.37 (14.31)	10.98	< 0.001^**^	53.31 (19.84)	46.64 (16.69)	6.66	< 0.001^**^
Dietary fiber (g / day)	5.59 (3.83)	4.64 (2.91)	0.94	< 0.001^**^	5.52 (3.17)	5.02 (3.03)	0.50	0.178
Calcium (mg / day)	415.39 (217.07)	352.55 (206.41)	62.85	0.005^*^	478.03 (270.82)	372.96 (194.01)	105.06	< 0.001^**^
Folate (mg / day)	60.13 (56.37)	53.88 (37.70)	6.26	0.131	60.62 (42.25)	60.08 (41.54)	0.53	0.925
Iron (mg / day)	13.35 (6.24)	11.36 (4.31)	3.21	< 0.001^**^	13.35 (6.24)	11.36 (4.31)	1.99	0.002^*^
Vitamin C (mg / day)	62.52 (59.60)	57.93 (53.02)	4.59	0.479	71.13 (65.97)	56.53 (53.44)	14.61	0.049^*^

^*^Significant at *p* value < 0.05; ^**^ Significant at *p* value < 0.001

Overall, both study groups showed a reduction in total energy intake, and macronutrients of carbohydrate, protein and fat intake over the six-month intervention period (*p* <  0.05). The reductions were significantly higher in the intervention group compared to the control group (*p* <  0.05) in carbohydrate (intervention = 28.50 g; control = 20.03 g), fat (intervention = 10.98 g; control = 6.66 g), protein (intervention = 9.33 g; control = 7.98 g) but similar reduction rate of total energy intake (224.36 g).

Apart from macronutrients, the analyses also showed significant reductions in dietary fibre, calcium, and iron over the six-month intervention phase in both study groups (*p* <  0.05). The intake of fibre was reduced in both study groups. However, the reduction was significant in the intervention group and not in the control group. Among all of the investigated micronutrients, only vitamin C and folate intake did not show a significant reduction over the 6 months of intervention phase in both study groups (*p* > 0.05).

Table 3 depicts the nutrient intake changes and its prediction to body fat loss in the intervention group. In the unadjusted linear regression analysis, the fibre, calcium and folate changes per 1000 kcal from the baseline were significantly associated with body fat changes. A decreased of fibre intake and calcium by one gram per 1000 kcal significantly reduced the body fat loss in the intervention group (*p* <  0.001). Meanwhile, the reduction of folate increased the body fat loss in that group (*p* <  0.05). No significant findings were observed in other nutrient changes. When the other possible confounding factors such as age, household income, baseline body fat and baseline of investigated nutrient per 1000 kcal were added in the regression model, reduction in fibre and calcium per 1000 kcal from the baseline significantly reduced the body fat loss (*p* <  0.05). The reduction of folate was no longer significant but the reduction in carbohydrate protein and fat per 1000 kcal intake were significantly associated with body fat loss increased (*p* <  0.05).

**Table 3:** Nutrient intake changes and its prediction to body fat changes during intervention period (0–6 months) in the intervention group

Nutrient intake changes per 1000 kcal ^a^	Body fat loss (kg) ^b^			
(0–6 months)	SLR	*p* value	MLR^c^	*p* value
	*b* (95% CI)		Adjusted *b* (95% CI)	
Carbohydrate (g/day)	−0.002 (− 0.005, 0.001)	0.184	0.012 (0.004, 0.019)	0.002^*^
Protein (g /day)	−0.012 (− 0.028,0.003)	0.125	0.039 (0.003, 0.074)	0.031^*^
Fat (g / day)	−0.006 (− 0.016, 0.004)	0.221	0.039 (0.013, 0.066)	0.004^*^
Dietary fiber (g / day)	−0.270 (− 0.407, 0.182)	< 0.001^**^	− 0.243 (− 0.470, − 0.017)	0.035^*^
Calcium (mg / day)	−0.003 (− 0.005, − 0.002)	< 0.001^**^	−0.004 (− 0.006, − 0.001)	0.006^*^
Folate (mg / day)	0.013 (0.002, 0.024)	0.024^*^	0.012 (− 0.002, 0.025)	0.090
Iron (mg / day)	−0.036 (− 0.091, 0.019)	0.194	0.094 (− 0.031, 0.219)	0.141
Vitamin C (mg / day)	0.001 (−0.005, 0.011)	0.436	−0.004 (− 0.015, 0.007)	0.471

^*^ Significant at p-value < 0.05; ^**^ Significant at *p*-value < 0.001

^a^Nutrient intake change: baseline nutrient per 1000 kcal – nutrient at 6 months per 1000 kcal

^b^Body fat change (body fat loss): baseline body fat mass – body fat mas at 6 months

^c^The model was adjusted for age, household income, baseline body fat and baseline investigated nutrient per 1000 kcal intake

The changes of nutrient intakes and its association with body fat loss in the control group were displayed in Table 4. Among all of the investigated nutrient components in unadjusted regression analysis, the reduction of fat and folate per 1000 kcal intake increased the body fat loss (*p* <  0.05). In the adjusted regression model, which included age, household income, baseline body fat, and baseline investigated nutrient per 1000 kcal, fat intake per 1000 reduction remained to increase the body fat loss (*p* <  0.05) in the control group. Other nutrient components showed no significant correlation with body fat loss.

**Table 4:** Nutrient intake changes and its prediction to body fat changes during intervention period (0–6 months) in the control group

Nutrient intake changes per 1000 kcal ^a^	Body fat changes (kg) ^b^			
(0–6 months)	SLR	*p* value	MLR^c^	*p* value
	*b* (95% CI)		Adjusted *b* (95% CI)	
Carbohydrate (g/day)	0.010 (−0.005, 0.024)	0.187	0.007 (−0.008,0.021)	0.364
Protein (g /day)	−0.012 (− 0.028, 0.003)	0.125	− 0.009 (− 0.057, 0.040)	0.728
Fat (g / day)	0.077 (0.036, 0.118)	< 0.001^**^	0.078 (0.032,0.125)	0.001^*^
Dietary fiber (g/day)	−0.008 (− 0.198, 0.182)	0.937	− 0.055 (− 0.285, 0.175)	0.636
Calcium (mg / day)	0.000 (− 0.004, 0.003)	0.811	0.000 (− 0.005, 0.004)	0.889
Folate (mg / day)	0.070 (−0.006, 0.019)	0.331	−0.013 (− 0.043, 0.016)	0.207
Iron (mg / day)	0.181 (0.058, 0.303)	0.004^*^	−0.074 (− 0.298, 0.150)	0.015^*^
Vitamin C (mg / day)	0.001 (−0.008,0.011)	0.791	0.001 (−0.010, 0.013)	0.846

^*^Significant at p-value < 0.05; ^**^ Significant at *p*-value < 0.001

^a^Nutrient intake change: baseline nutrient per 1000 kcal – nutrient at 6 months per 1000 kcal

^b^Body fat change (body fat loss): baseline body fat mass – body fat mas at 6 months

^c^The model was adjusted for age, household income, baseline body fat and baseline investigated nutrient per 1000 kcal intake

## Discussion

The current study showed a significant reduction in body fat mass after the six- months intervention period in both study groups. The dietary intake over the period of 6 months demonstrated a significant reduction in total energy, macronutrients, dietary fibre (in the intervention group) and some of the minerals such as calcium and iron. Weight loss intervention characterized by intensive frequent contact in this study might be the influential factor for dietary intake reduction. Frequent study contact has been observed to support the weight loss achievement as observed in other intervention studies [17, 18]. A general health seminar, which had been delivered in the control group might have influenced their awareness of nutrient intake and be more accountable for the health behaviour [18]. This is consistent with behavioural theory whereby people who have formed actions plans were more likely to be successful compared to those merely considering a goal [19].

It has to be noted that one of the components of the dietary package received by both the study groups included self-monitoring of their dietary intake. This empowerment factors might have supported the participants to control and comply with the diet they took. Engagement in routine self-monitoring tasks such as recording of food intakes could promote weight loss action due to the enhancement of self-awareness [20, 21].

Reduction in intakes of calories, macronutrients, fibre and other nutrients were higher in the intervention group compared to the control group although both groups showed a significant reduction at the end of the intervention. The intervention group received supervised dietary counselling by the trained dieticians/nutritionists, while the control group did not have any access to dietary intervention except self-monitoring. The findings suggested that dietary counselling tailored to the individualized needs and intakes were proven to cut down the overall energy and fat intake due to better compliance with the prescribed diet. Supervised dietary interventions allow participants to receive advice, knowledge, motivation, and feedback from health care providers that ultimately increase their self-efficacy [22].

Despite the reduction of energy and macronutrients intake, the intervention and control group experienced a significant reduction of essential nutrients including fibre, calcium, iron, folate and vitamin C. This unwanted side effect warranted important precautions that need to be highlighted when prescribing low-calorie diets. It is important to emphasize the adequacy of the essential nutrients when consuming low-calorie diet. Several studies had shown a micronutrient intake reduction was accompanied by a low-calorie diet focusing on macronutrient intakes [23]. Inadequate mineral might alter nutrient metabolism [24] and increase obesity risk [25].

Our findings demonstrated that a reduction in each gram of protein, carbohydrate and fat per 1000 kcal from the baseline increased the body fat loss to 12 g (carbohydrate per 1000 kcal) and 39 g (protein and fat per 1000 kcal) after adjusting for the potential confounding factors in the in the intervention group. In the control group, a reduction in each gram of fat per 1000 kcal significantly increased the body fat loss to 78 g. Restriction of energy intake is the main method to create negative energy balance that leads to weight loss. In this study, the nutrient changes were calculated per 1000 kcal intake to limiting the influence of energy intake as the primary factor in energy balance. The significant effects of macronutrient reduction to the body fat loss had been reported in the other studies [26, 27]. Macronutrients have different metabolic roles in energy homeostasis including affect metabolism, appetite, and thermogenesis although the diet contains similar energy amount [27].

Although there was a calorie reduction in both study groups, the proportion of carbohydrate, protein, and fat at the end of intervention were still within the recommended intake in the intervention group. It has been proposed that the diet quality may have different metabolic effects and energy partitioning unique to calorie intake [28]. For instance, the intake of carbohydrate with low glycaemic load and high intake of omega 3 in dietary fat is suggested to increase post-prandial energy expenditure [29–31].

As for the protein intake, although its intake was considered within the standard range (15–20% of energy), in contrast to a high protein diet (25–35% of energy) that has been successfully associated with weight loss [27] its correlation to body fat reduction suggested the confounding effect of food selection and overall diet compositions. These factors, were not controlled in the analysis (e.g., low-fat milk versus full-fat milk or red meat versus white meat) might have a similar protein per serving but have a different fat content or quality of the diets. This was supported in the intervention group where there was a significant reduction in saturated fat content that might be originated from animal fat (baseline saturated fat = 10.47 (7.42) g; end intervention saturated fat =8.78 (4.13) g, *p* < 0.05). Total fat oxidation in saturated fat was found to be lower compared to monounsaturated and polyunsaturated fat intake [32], hence, induced weight loss and body fat loss [33].

Observation in the control group showed the reduction in dietary fat was associated with body fat loss (*p* < 0.05). Given these results, it was confirmed that decreasing total dietary fat intake was associated with decreases in body weight measure as presented in the other study [34]. The reduction of protein and carbohydrate in the control group, however, was not associated with body fat loss as observed in the intervention group. As previously highlighted, the role of macronutrients and the quality of the diet has a different metabolic effect in energy balance although the calorie amount is similar.

Apart of those factors, it was acknowledged that the method for measuring dietary intake to assess the fat, carbohydrate, and protein intake is inherently inaccurate and imprecise, as it relies on self-report and might be biased [35]. In addition, unsupervised dietary intervention in the control group might lead to poor compliance. The diminished effect of all macronutrients to body fat loss after confounding factor adjustments suggests a stronger effect of other factors such as income, and other socio-demographic factors. In the control group, income remained a significant confounder for body fat loss in the adjusted regression analysis.

The reduction of fibre intake significantly reduced the body fat loss in the intervention group. Each gram reduction of fibre per 1000 kcal intake reduced 243 g of body fat loss (*p* = 0.035). In another study, the dietary fibre had an independent association with body weight after multiple factors adjustment [36]. Fibre is suggested to increase the satiety effect, decreasing energy density and energy intake that involve in weight management [37]. It also useful in enhancing compliance to a energy-restricted diet by reducing hunger [38]. Energy digestibility has been shown to decrease by 3–4% following 20–25 g / d increases of fibre intake [39]. The fibre intake in both study groups, however, did not meet the minimum recommendation of 20 g / day or 50% of the requirements. The previous study has demonstrated an intake of whole grain as the indigestible fibre had an inverse relationship to BMI and reduced abdominal fat [40]. Compared with intervention group, insoluble fibre in the control group during the study was lower (Control: Baseline = 0.38 (0.63) g, 6-month = 0.48 (0.84) g; Intervention: Baseline 0.52 (1.11) g, 6- month =0.56 (0.98) g). This might explain the discrepancy of the correlation between the fibre changes and body fat loss in the control group.

Apart from dietary fibre, accumulating evidence suggest that calcium-rich diets play direct roles in the prevention and treatment of obesity due to its property in Ca^2+^ in regulating adipocyte metabolism [41, 42]. In the intervention group, the reduction of calcium per 1000 kcal significantly reduced the body fat loss. Each gram reduction of calcium intake reduced 4 g of body fat loss. However, the calcium intake in the intervention group was lesser than the recommended nutrient intake, thus, it might suggest the reason of the small change to body fat loss when calcium intake was reduced. In the control group, the effect on calcium to body fat changes was not observed. Melanson et al. [43] had concluded that increasing dairy/calcium intake increased body fat oxidation was demonstrated under energy deficit. The energy intake in the control group was slightly higher than in the intervention group; in addition, the prescription for calorie intake in the control group was not determined specifically during the intervention.

Adherence to weight loss nutrient requirement was the challenges to the free-living study participants with the economy disadvantage. Although in the changes of diet in the intervention group showed promising findings, the changes were small. We acknowledged that self-reported diet intake may likely lead to underreporting of energy and nutrient intake. This study also did not evaluate the association of physical activity, which could induce the body fat loss.

Despite these shortcomings, the viability of this study was boosted by the good retention rate, which was 20.7% of attrition by 6 months of the study period. By implementing various methods to increase the representation of the participants during the study period, successful engagements with the study contacts we able to be maintained. However, further consideration must be given to cost and the additional time that will add to the study. The other strength of this study was the ability to investigate the association of discrete nutrients with body fat loss by reporting the changes of nutrients by 1000 kcal, which, limits the covariate effect of total energy intake as the primary factor in energy balance.

## Conclusions

The results of this study indicate that increasing fibre and calcium, and reducing carbohydrate, fat and protein as part of a calorie-controlled diet, helped body fat loss in the intervention group. Nonetheless, the lack of effect of nutrient changes in the control group except for fat and iron suggests that the quantity of diet was not the only factor influencing body fat loss. There were other components such as compliance, and proper nutrition guidance that were missed in the controlled group. Therefore, emphasizing those factors when recommending low calorie diets to free-living, economically disadvantaged community dwellers would be beneficial.

## Abbreviations

BIA: Bioelectrical impedance analyser
BMI: Body Mass Index
Ca^2+^: Calcium
MyBFF@home: My Body Fit and Fabulous at home
